# A Mutant of SWAP-70, a Phosphatidylinositoltrisphosphate Binding Protein, Transforms Mouse Embryo Fibroblasts, Which Is Inhibited by Sanguinarine

**DOI:** 10.1371/journal.pone.0014180

**Published:** 2010-12-02

**Authors:** Yasuhisa Fukui, Sayoko Ihara

**Affiliations:** 1 Laboratory of Signal Transduction, Hoshi University, Tokyo, Japan; 2 Institute of Cellular and Systems Medicine, National Health Research Institutes, Zhunan, Taiwan, Republic of China; 3 Graduate School of Agricultural and Life Sciences, University of Tokyo, Tokyo, Japan; Universidade Federal do Rio de Janeiro, Brazil

## Abstract

SWAP-70, a phosphatidylinositol trisphosphate (PtdIns(3,4,5)P_3_) binding protein, has been suggested to be involved in transformation of mouse embryo fibroblasts (MEFs) as well as membrane ruffling after growth factor stimulation of the cells. A mutant, SWAP-70-374, was found to be able to bind to F-actin *in vitro*, whereas wild-type SWAP-70 failed to do so. This mutant was present at the plasma membrane without any stimulation while the wild-type protein was present only in the cytosol unless cells were stimulated with EGF. Expression of this mutant in MEFs resulted in morphologic transformation, fast growth, and loss of contact inhibition, suggesting that SWAP-70 with this mutation can transform the cells. ERK1/2 was activated in SWAP-70-374-transformed cells. Use of MEK inhibitors revealed that the ERK1/2 pathway does not affect the cell growth of MEFs but is responsible for loss of contact inhibition. To investigate the function of SWAP-70 further, drugs that can inhibit SWAP-70-dependent cell responses were screened. Among various drugs, sanguinarine was found to inhibit transformation of MEFs by SWAP-70-374. This drug was able to inhibit SWAP-70-mediated membrane ruffling as well, suggesting that its effect was closely related to the SWAP-70 signaling pathway. These results suggest that SWAP-70-374 can activate some signaling pathways, including the ERK1/2 pathway, to transform MEFs.

## Introduction

SWAP-70 is a phosphatidylinositol trisphosphate (PtdIns(3,4,5)P_3_) binding protein, that has been implicated to play a role in the formation of cancer. Strong expression of SWAP-70 is often seen in human B-cell neoplasms [1]. SWAP-70 has been also shown to be expressed in higher levels in malignant gliomas than in low-grade ones or normal brain tissue [2]. These results suggest that SWAP-70 may be closely related to formation of malignant tumors *in vivo*. Involvement of SWAP-70 in transformation of the cells has also been suggested *in vitro*. We have shown that SWAP-70 is required for the anchorage-independent growth of v-Src-transformed MEFs [3]. In addition, growth of the cells lacking SWAP-70 has been shown to be slower than that of the cells expressing SWAP-70 [3]. These results suggest that SWAP-70 may be involved in regulation of cell growth in some way. It has been suggested that SWAP-70 is important for cell motility and invasion of the tumor cells [2], [4].

Although how SWAP-70 contributes to the formation of cancers is largely unknown, some of SWAP-70's activities at the biochemical or cell biological level have been revealed. SWAP-70 contains a pleckstrin homology (PH) domain, which is responsible for PtdIns(3,4,5)P_3_ binding, in the central part and a coiled-coil domain in the carboxyl-terminal half [5], [6]. In addition, carboxyl-terminal region of SWAP-70 has been shown to bind to non-muscle F-actin *in vitro* [7]. One of the well-studied cell responses related to actin rearrangement is membrane ruffling, which is closely related to cell motility. SWAP-70 translocates from the cytoplasm to the membrane ruffles upon PtdIns 3-kinase activation after growth factor stimulation and co-localizes with F-actin in adherent cells such as MEF or Cos7. Cells lacking SWAP-70 show impaired membrane ruffling after growth factor stimulation, suggesting that SWAP-70 may play a crucial role in induction of membrane ruffling [5]. SWAP-70 lacking the F-actin binding domain has been shown to act as a dominant negative reagent for membrane ruffling, suggesting that this actin-binding activity is important for membrane ruffling [7]. Binding of SWAP-70 to activated Rac1, which has been shown to regulate actin rearrangement including membrane ruffling, has been also detected [7]. Taken together with the fact that SWAP-70 binds to PtdIns(3,4,5)P_3_, a product of PtdIns 3-kinase, that has been also suggested to be essential for membrane ruffling, it is likely that SWAP-70 is an important molecule that may put the functions of PtdIns(3,4,5)P_3_, F-actin, and Rac1 together. Supporting these findings, SWAP-70 has been shown to be essential for proper homing of B cells to lymphoid organs, which may require F-actin rearrangement [8]. Because F-actin rearrangement is likely to be related to cell transformation, these findings support the idea that SWAP-70 contributes to tumor formation in some way.

Sanguinarine, a benzophenanthridine alkaloid, has been shown to exhibit anti-cancer activity *in vivo* and *in vitro* [9], [10], [11], [12], [13], [14], [15]. For instance, sanguinarine exhibits antiproliferative and antiangiogenic effects in melanoma and prevention activity of occurrence of skin cancers. There are also a number of reports suggesting that sanguinarine inhibits growth of tumor cell lines and induces apoptosis. Recently, it has been suggested that sanguinarine interacts with DNA and histones, which might be the mechanism for its anti-tumor activity [16]. However, this does not completely explain the fact that sanguinarine is effective only for certain tumor cell lines.

In this paper, we demonstrate that a mutant of SWAP-70 can transform mouse embryo fibroblast and further suggest that an anti-cancer drug, sanguinarine inhibits SWAP-70-dependent cell responses.

## Materials and Methods

### Cells and culture conditions

Mouse embryo fibroblasts (MEFs) were cultured from a 129/SvEMS strain in Dulbecco's modified minimal essential medium (DMEM) supplemented with 10% fetal bovine serum. The culture was maintained carefully and established as an immortalized cell line: this was named as MEF clone 18. However, MEFs are usually mixtures of cells derived from various origins: thus cells can give various phenotypical backgrounds. For this reason, when cell lines expressing some gene are produced, each line could have a different background. To deal with this problem, cells were isolated by limiting dilution method and grown from single cells. One of these cells, 18-2, was used in this study [3]. In this way, phenotypic background should be identical among the clones. 70-5 is a MEF cell line that expresses wild-type SWAP-70 [3].

Cos7 cells were cultured in DMEM supplemented with 5% calf serum and mutant SWAP-70 genes cloned into pEGFP-C1 (Clontech Inc., Madison, WI), an expression vector, were introduced into these cells by electroporation [17].

### Establishment of cell lines carrying the exogenous SWAP-70 genes

To obtain MEF clones expressing human mutant SWAP-70s, an expression vector pMIKHyg harboring wild-type or mutant SWAP-70 was used. As has been described previously, pMIKHyg, an expression vector, contains the hygromycin-resistant gene instead of the G418-resistant gene in pMIKNeo, which has been described before [3]. SWAP-70-374 carries two point mutations K374A/K375A, which was introduced using a primer, 5′-gcagcagaagaggaagcggcgcgccttcagactcaa-3′, by the method described by Sawano et al. [18]. SWAP-70-374m1 carries additional mutations within the PH domain of SWAP-70, K219A/K220A, which abolish the binding activity of SWAP-70 to PtdIns(3,4,5)P_3_ [19].

20 µg of DNA was introduced into about 3×10^6^ cells by electroporation using Cell Porator (Bethesda Research Laboratories, Bethesda, MD) at 225 V with 800 µF capacitance. The stable transformants were established by selection of the cells with 10 µg/ml hygromycin (Wako Co. Ltd., Tokyo). SWAP-70-374-2 and SWAP-70-374-24 cells were obtained as SWAP-70-374 protein-expressing cells and SWAP-70-374m1-12 and SWAP-70-374m1-14 cells as SWAP-70-374m1-expressing cells.

### Antibodies and Western blotting

Anti-SWAP-70 antibody was raised against bacterially expressed full-length human SWAP-70 fused to glutathione-S transferase as described before [3]. This antibody does not recognize mouse SWAP-70 but does recognize human SWAP-70. Anti-ERK1/2 and anti-phospho-ERK1/2 antibodies were purchased from Cell Signaling Technology Inc. (Danvers, MA). Anti-β-actin antibody was from Sigma-Aldrich (St. Louis, MO).

Western blotting was done as described before [3]. Briefly, blocking was done with 1% skim milk, and the protein was visualized by the ECL system.

### Determination of cell growth

Cells were plated on the 6-cm dishes at a density of 1×10^5^ cells per dish and maintained with medium change every other day. The numbers of the cells per dish were counted. For examination of cell survival, cells were plated at a density of 2×10^5^ cells per 6-cm dish and maintained without medium change. The cell numbers shown in the figures are the means of three independent experiments. Sanguinarine was purchased from Extrasynthese (Genay Cedex, France), PD98059 and U0126 were from Wako Co Ltd.

### F-actin cosedimentation assay

Binding of SWAP-70 WT/374 to F-actin was tested as described before [7]. Briefly, human non-muscle actin (Cytoskeleton Inc., Denver, CO) was polymerized in F-buffer (50 mM KCl, 2 mM MgCl_2_, 0.2 mM ATP, and 0.2 mM DTT in 2 mM imidazole, pH 7.1) at room temperature for 30 min and incubated with purified recombinant wild-type His-SWAP-70 or His-SWAP-70-374 for 1 hr at room temperature. After ultracentrifugation at 109,000×g for 30 min at 25°C, the proteins in the supernatant or the pellet were analyzed by SDS-PAGE, which was followed by Coomassie Blue staining. The amount of SWAP-70 in each lane was quantified by densitometric analysis using ImageJ 1.43 u (National Institutes of Health, USA). The ratio of amount of SWAP-70 in the pellet to that in the supernatant was calculated in each sample. Then the value of the sample with F-actin was normalized to that of the sample without F-actin. The results show the relative amount of SWAP-70 specifically bound to F-actin. The numerical formula to obtain the results is: (Relative affinity)  =  {(amount of SWAP-70 in the pellet in the experiment with F-actin)/(amount of SWAP70 in the supernatant in the experiment with F-actin)}/{(amount of SWAP-70 in the pellet in the experiment without F-actin)/(amount of SWAP70 in the supernatant in the experiment without F-actin)}.

### Confocal microscopy

Cells were fixed with 3.7% formaldehyde for 5 min at room temperature. After permeabilization of the cells with PBS containing 0.1% Triton for 5 min, F-actin was stained with TRITC-phalloidin (Sigma-Aldrich). Cells were analyzed using a confocal fluorescence microscope (Fluoview 300, Olympus, Tokyo).

### PtdIns(3,4,5)P_3_ analogue bead-binding assay

Proteins in 293T cells transfected with expression vectors for the wild-type and the mutant SWAP-70s fused with GFP were extracted by freezing-thawing three times in phosphate-buffered saline (PBS) containing 1 mM EDTA, and 1 mM PMSF. After removal of cell debris by ultracentrifugation (100,000×*g*, 30 min), the samples were mixed with PtdIns(3,4,5)P_3_ analogue beads. Proteins bound to the beads were eluted with an SDS sample buffer and analyzed by Western blotting with anti-GFP antibody.

### Soft agar colony formation assay

1×10^5^ cells were suspended in 2 ml of a medium containing 0.2% agarose and plated onto a basal agarose layer consisting of a medium containing 0.5% agarose in 6-cm dishes and incubated for 2 weeks. 2 ml of a medium containing 0.2% agarose was added onto the culture every week.

### Serum requirement for the cell growth

Cells were plated at a density of 5×10^4^ cells per 6-cm dish and cultured for 4 days in the medium containing low serum with a medium change on day 2. Growth of the cells were observed under the microscope.

## Results

### SWAP-70-374 localizes at the plasma membrane

**Figure 1:**
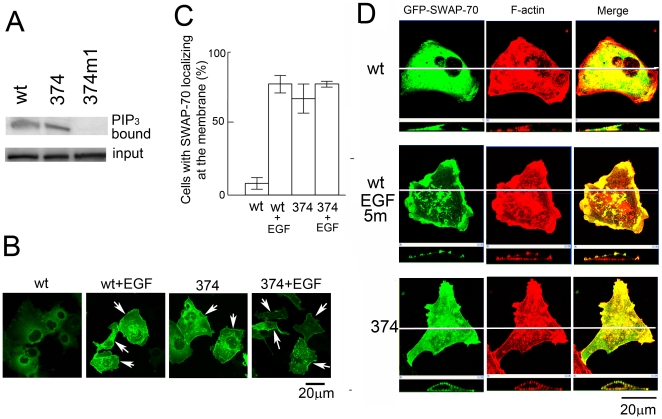
A mutant SWAP-70, SWAP-70-374, locates at the plasma membrane. A, Binding activities of the mutant SWAP-70s to PtdIns(3,4,5)P_3_ were analyzed using the bead binding assay. The upper panel shows SWAP-70s bound to the beads and the lower panel shows the protein used for the assay. B, Expression vectors for the wild-type while the mutant SWAP-70 were introduced into Cos7 cells and the localization of expressed proteins was analyzed by confocal microscopy. The stacked images are shown. The arrows indicate the cells whose SWAP-70 were found at the plasma membrane. C, The percentages of the cells whose GFP-SWAP-70s were found at the plasma membrane are shown. Experiments were repeated three times and the means are shown in the graph. The error bars show standard deviation. D, Expression vectors for GFP-SWAP-70 or GFP-SWAP-70-374 were introduced into Cos7 cells by eletroporation. The cells expressing GFP-SWAP-70 were stimulated with EGF (100 ng/ml) for 5 min. After fixation with formaldehyde, cells were stained with TRITC-phalloidin and observed using confocal microscopy. The upper panels show XY images of the cells and the lower XZ images. The lines in the top panels show the place where XZ images were made.

We introduced various mutations in SWAP-70 to find out interesting mutants. Especially, charged amino acids were targeted because they might affect the conformation of the protein. Among them, the 374 (K374A/K375A) mutation, which resides just downstream of the central PH domain, showed an interesting phenotype. This mutation did not affect the PtdIns(3,4,5)P_3_ binding activity of the protein (Fig. 1A). Cellular localization of the protein was examined using green fluorescence protein (GFP) as a marker. Cos7 cells were transfected with the expression vectors for wild-type and mutant SWAP-70s fused to GFP. When the proteins are in the cytosol, the signals of GFP get gradually stronger toward the nuclei and the nuclei look almost transparent. In contrast, when the proteins are located at the plasma membrane, the strength of the signals is even and the nuclei look turbid. By these criteria, localization of SWAP-70s was observed under the microscope (Fig. 1B). The wild-type SWAP-70 was found mostly in the cytosol in unstimulated cells: but it moved to the plasma membrane and accumulated at the membrane ruffles after stimulation with EGF as described before (Fig. 1B). In contrast, a mutant, SWAP-70-374, was found at the plasma membrane in most of the cells even when cells were not treated with EGF (Fig. 1B). The protein moved to the membrane ruffles after stimulation with EGF, suggesting that this mutant protein is functional (Fig. 1B). The percentages of the cells with SWAP-70 located at the plasma membrane are shown in Fig 1C. This was confirmed by examining the X-Z image of the cells. As shown in Fig. 1D, wild-type protein found in the cytosol in unstimulated cells moved to the membrane after stimulation by EGF. The mutant, 374, also localized at the plasma membrane even without EGF-stimulation. It should be noted that this mutant co-localized with F-actin.

### Biochemical properties of SWAP-70-374

**Figure 2:**
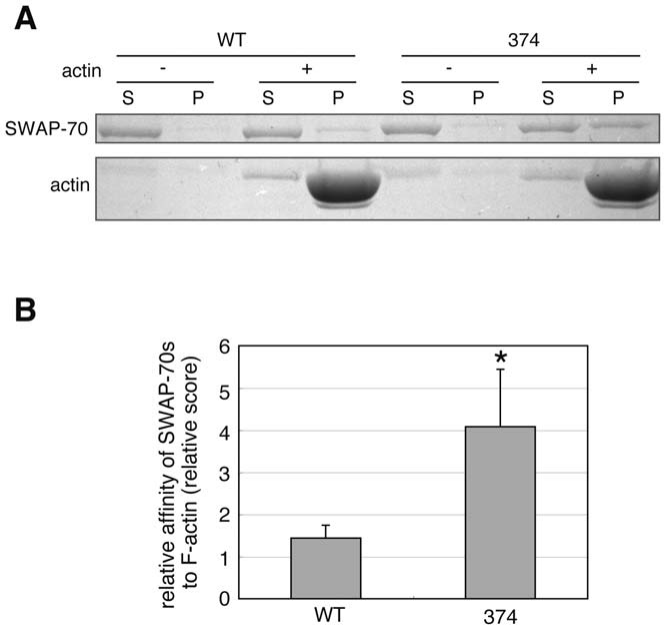
F-actin co-sedimentation assay of SWAP-70s. A, F-actin binding activity of SWAP-70s was examined. His-SWAP-70 (WT) or His-SWAP-70-374 (374) was mixed with (+) or without (−) 20 µM polymerized non-muscle actin. After incubation for 1 hr, samples were ultracentrifuged. Proteins in the supernatant (S) and the pellet (P) were analyzed by SDS-PAGE followed by CBB staining. The faint bands seen at the position of actin in the lane of actin (−) are the degradation product of His-SWAP-70-WT/374. B, The same experiments as in A were done for five times and the amount of SWAP-70 in each lane was quantified. Relative affinity of SWAP-70s to F-actin were calculated as described in Materials and Methods. Results from five independent experiments are presented as the means. The error bars indicate standard deviation (* P<0.02; t-test).

Because SWAP-70-374 co-localized with F-actin, its binding activity to F-actin was analyzed. As shown previously, SWAP-70 exhibits actin binding ability in a co-sedimentation assay with F-actin only when the C-terminal portion containing actin binding domain was used. In contrast, full-length SWAP-70 does not exhibit this ability [7]. Considering the fact that SWAP-70 co-localizes with F-actin only when the cells are stimulated, these results suggest that the actin binding domain of full-length SWAP-70 may be masked and may be exposed only when activated. Because SWAP-70-374 co-localizes with F-actin under unstimulated conditions, we asked whether SWAP-70-374 bound to F-actin in its full-length form *in vitro*. The co-sedimentation assay was performed with non-muscle F-actin using two purified recombinant proteins: His-SWAP-70-WT and 374. As shown in Fig. 2A, a considerable amount of His-SWAP-70-374 co-sedimented with F-actin, whereas only very small amount of His-SWAP-70-WT co-sedimented. To quantify the difference between the F-actin binding ability of the two proteins, the intensity of the bands of SWAP-70s was measured and the scores that indicate relative affinity of the proteins to F-actin were calculated (Fig. 2B). His-SWAP-70-374 showed much higher affinity to F-actin than His-SWAP-70-WT. The small amount of His-SWAP-70-WT co-sedimented with F-actin could be attributed to the nonspecific binding with F-actin through PH domain as previously reported [7]. These results suggest that SWAP-70-374 may take the activated conformation capable of binding to F-actin.

### Elements required for translocation of SWAP-70-374 to the membrane

**Figure 3:**
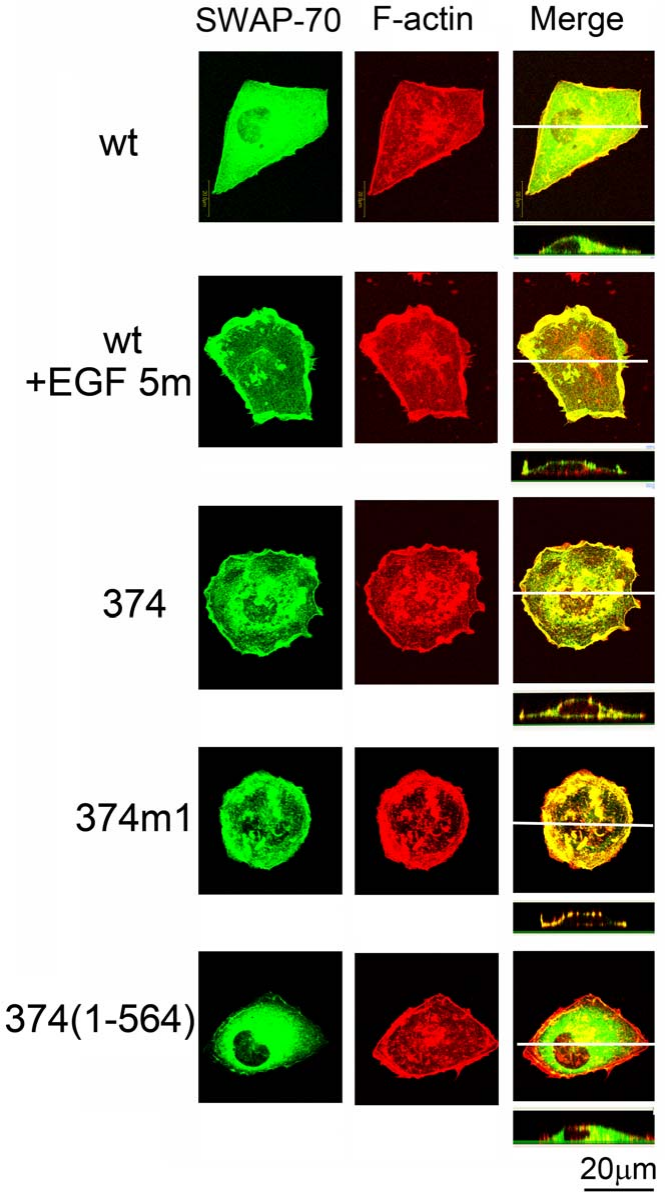
Localization of mutant SWAP70s. Expression vectors for GFP-SWAP-70, GFP-SWAP-70-374, GFP-SWAP-70-374m1, and GFP-SWAP-70 (1-564) were introduced into Cos7 cells by eletroporation. The cells expressing GFP-SWAP-70 were stimulated with EGF (100 ng/ml) for 5 min. After fixation with formaldehyde, cells were stained with TRITC-phalloidin and observed under the confocal microscope. The upper panels show XY images of the cells, while the lower panels show XZ images. The lines in the top panels show the position where XZ images were made.

How SWAP-70-374 localized at the plasma membrane was examined using the mutants containing additional mutations. As shown in Fig. 3, SWAP-70-374 was found at the plasma membrane in most of the cells as mentioned above. Because translocation of SWAP-70 to the plasma membrane after growth factor stimulation requires binding of SWAP-70 to PtdIns(3,4,5)P_3_ through its PH domain [19], dependence of localization on the PH domain- PtdIns(3,4,5)P_3_ interaction was examined using a mutant SWAP-70-374m1 carrying an additional mutation in the PH domain, which abolishes the PtdIns(3,4,5)P_3_ binding activity (Fig. 1A). SWAP-70-374m1 was still found at the plasma membrane, suggesting that the binding activity to PtdIns(3,4,5)P_3_ was not required for translocation of the protein carrying the 374 mutation. In contrast, the SWAP-70-374 (1-564) protein, which lacked the F-actin binding domain at the very carboxyl-terminal region [7], failed to translocate to the plasma membrane, suggesting that the F-actin binding activity was driving the protein to the plasma membrane. These observations were reproducibly seen in all of the cells expressing the SWAP-70 mutants.

### Morphologic transformation of MEFs by expression of the mutant SWAP-70

Because SWAP-70 is required for full transformation of MEFs by the v-Src oncogene [3], it is possible that activated SWAP-70 can contribute to transformation of MEF 18-2. To examine this possibility, the mutant protein, SWAP-70-374, was expressed in MEFs. SWAP-70-374m1 was also used.

**Figure 4:**
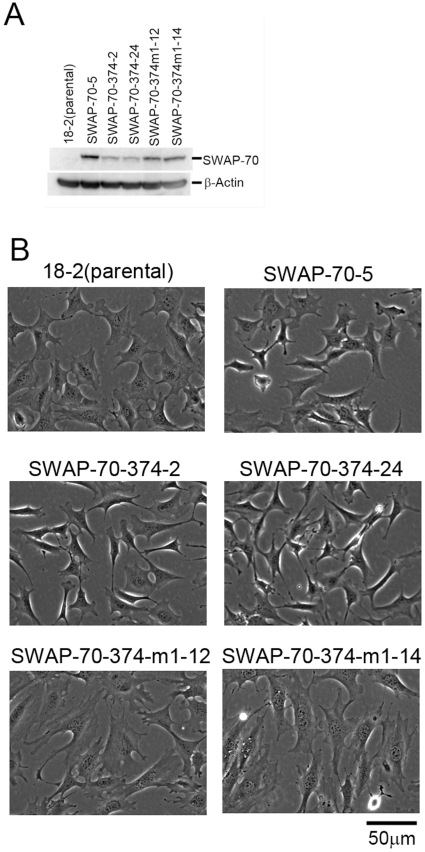
Morphologic transformation of MEFs expressing exogenous SWAP-70. A, Western blotting analysis of the exogenously expressed SWAP-70. Western blotting of SWAP-70 was done using cell lysate from each line (30 µg protein) (upper panel). β-actin was also detected as a control (lower panel). B, Cell morphology of MEFs expressing exogenous SWAP-70s. Cells were plated at a density of 3×10^5^ cells in 6-cm dishes. After cultivation for 1 day, cell morphology was observed under the microscope.

Expression vectors for the SWAP-70s were introduced in the MEFs by electroporation and the stable transformants were selected by hygromycin. As shown in Fig. 4A, MEF clones expressing mutant SWAP-70s were obtained. When cells were plated in a low density, morphology of the cells expressing SWAP-70-374 was shorter and thicker than that of the wild-type cells, suggesting that expression of SWAP-70-374 weakens the ability of the cells to attach to the substratum as is the case with transformation by the *src* oncogene (Fig. 4B) [3]. In contrast, cells expressing SWAP-70-374m1 were flatter than the wild-type cells, suggesting that they are strongly attached to the substratum (Fig. 4B).

### Expression of SWAP-70 mutants affect the cell growth

**Figure 5:**
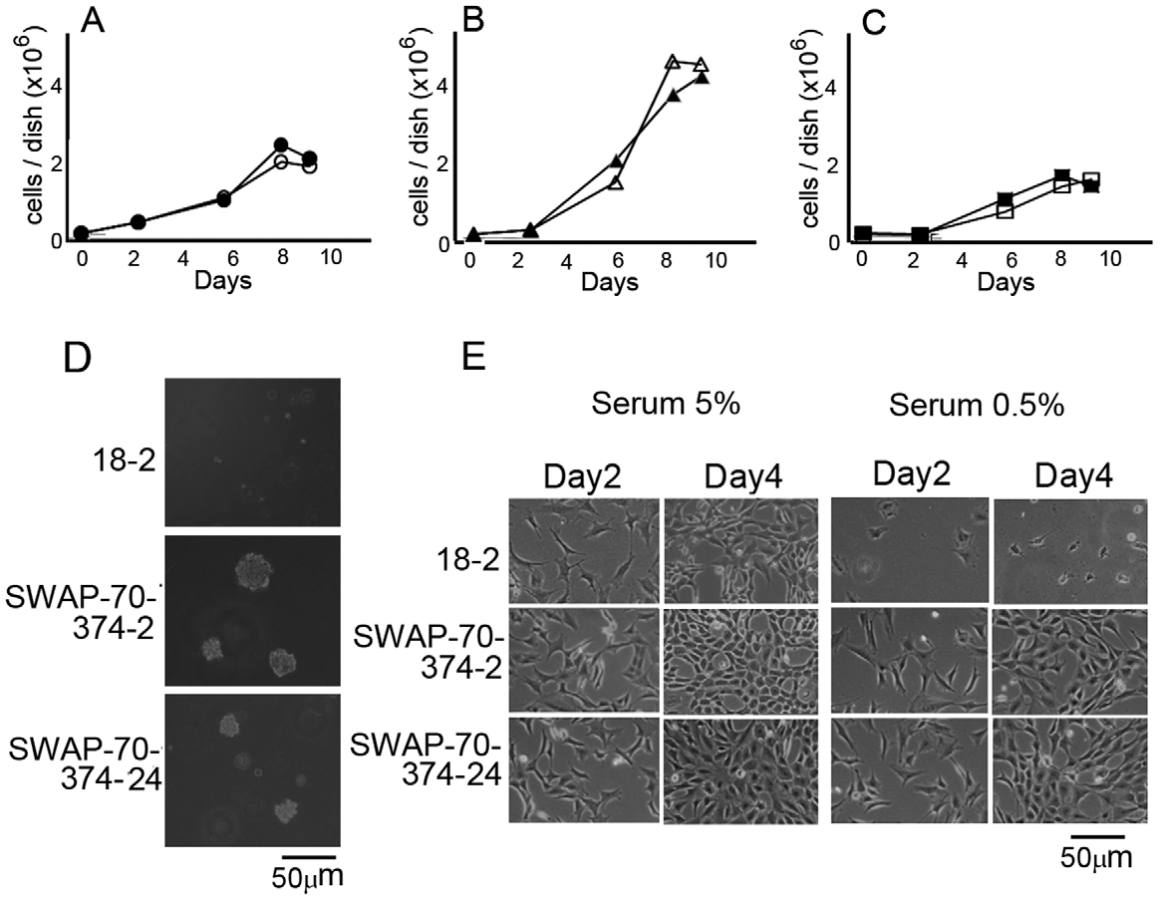
Growth of MEFs expressing exogenous SWAP-70s. Cells were plated at a density of 1×10^5^ cells in 6-cm dishes. A–C, Numbers of cells in each dish were counted. Medium was replaced every other day. A, parental MEFs (closed circles) and MEFs expressing wild-type SWAP-70, SWAP-70-5, (open circles) are shown. B, MEFs expressing SWAP-70-374: SWAP-70-374-2 (closed triangles) and SWAP-70-374-24 (open triangles). C, MEFs expressing SWAP-70-374m1: SWAP-70-374m1-12 (closed squares) and SWAP-70-374m1-14 (open squares). The standard deviations for each point were less than 4%. D, Soft agar colony formation assay was performed as described in Materials and Methods. After cultivation for 18days, the colonies were observed under the microscope. E, Cells were plated at a density of 5×10^4^ cells per 6-cm dish in the medium containing 5% or 0.5% fetal bovine serum. Images of the cells after cultivation for 2 or 4 days are shown. As shown in the figure, parental cell line did not grow in the medium containing 0.5% fetal bovine serum, whereas growth of SWAP-70-374-2 and SWAP-70-374-24 was not greatly affected.

Cell growth of the transformants was monitored. MEFs expressing SWAP-70-374 grew faster than the parental cells or the cells expressing wild-type SWAP-70 (Fig. 5A and B). The growth curve differed from time to time depending on the slight difference in the numbers of the cells plated on the cells, reflecting the delicate nature of the cell lines. However, in any case, growth rates of SWAP-70-374-expressing cells were higher than those of control cells. Also, saturation density of the cells was much higher in the cells expressing SWAP-70-374 (compare Fig. 5A and B). Although the growth rate of the cells was greatly enhanced and it appeared that cell number would increase without limit, these cells never piled up but rather formed a dense monolayer, unlike many other transformed cells, such as Src-transformed MEFs. In contrast, those expressing SWAP-70-374m1 grew more slowly than the parental cells with low saturation density (compare Fig. 5 A and C). These results agree with the fact that cells were flatter than the parental ones and suggest that SWAP-70-374m1 has an inhibitory effect on cell growth.

Whether expression of SWAP-70-374 induces oncogenic transformation was also tested by the soft agar colony formation assay. As shown in Fig. 5D, the SWAP-70-374-expressing cells formed colonies in soft agar, whereas parental cells did not.

Many of the malignant transformants can grow under the low-serum conditions. This was also tested. As shown in Fig. 5E, when the serum concentration was decreased to 0.5%, parental cells did not grow. In contrast, the growth of the SWAP-70-374-expressing cells was not affected. Growth rate in the medium containing 0.5% serum was about 80% of that in the medium containing 5% serum. These results suggest that SWAP-70-374 can induce malignant transformation. The focus formation did not give clear results. Because SWAP-70-374-transformed cells grow in monolayer, it was very hard to detect the foci. The SWAP-70-374-transformed cells just pushed other cells to expand their area (data not shown).

As shown in Fig. 4A, the expression levels of the exogenous SWAP-70 were always low in SWAP-70-374 and relatively higher in SWAP-70-374m1. The reason for this is not known. The low expression of SWAP-70-374 could be due to its toxicity or its instability.

### MEFs expressing SWAP-70-374 are sensitive to nutrient starvation

**Figure 6:**
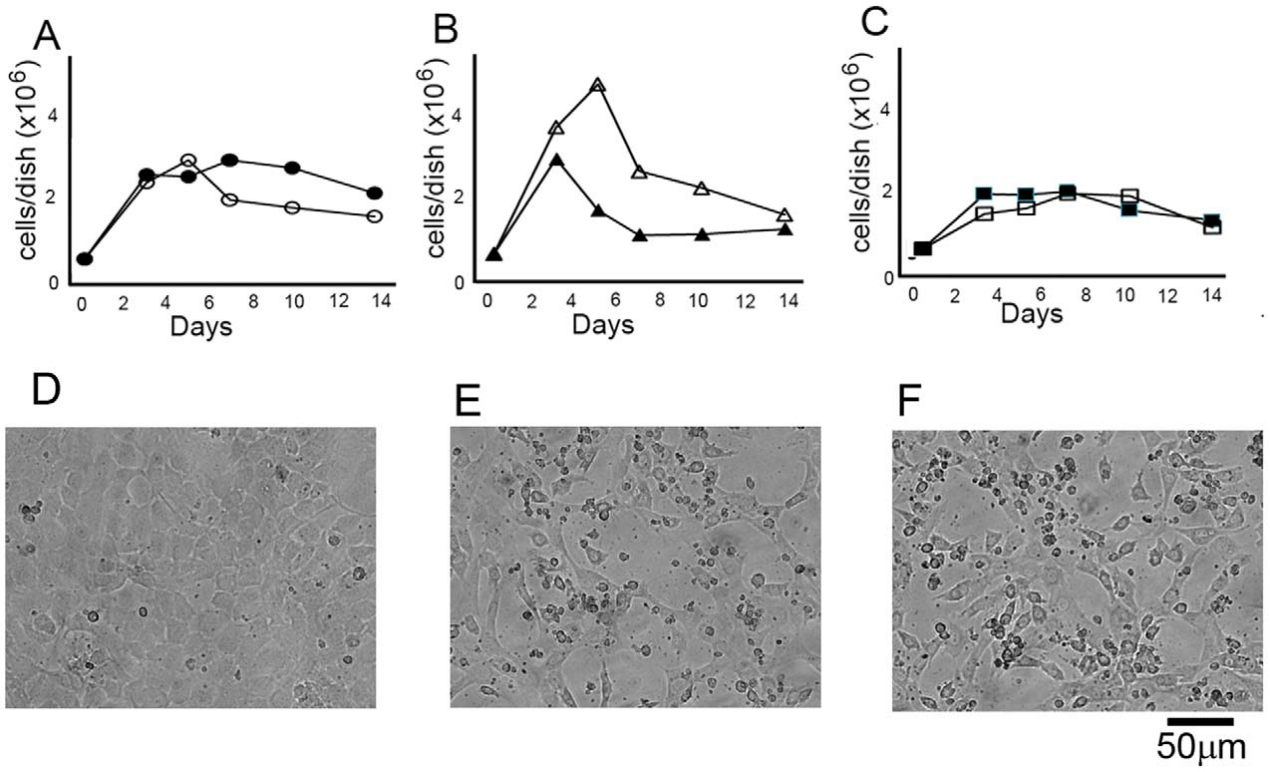
Tolerance of MEFs expressing exogenous SWAP-70s to nutrient starvation. Cells were cultured at a density of 2×10^5^ cells in 6-cm dishes and cultured without medium replacement. A–C, Numbers in each dish were counted. D–F, Photographs of the cells when nutrient starved. A, parental MEFs (closed circles) and MEFs expressing wild-type SWAP-70, SWAP-70-5, (open circles) are shown. B, MEFs expressing SWAP-70-374: SWAP-70-374-2 (closed triangles) and SWAP-70-374-24 (open triangles). C, MEFs expressing SWAP-70-374m1: SWAP-70-374m1-12 (closed squares) and SWAP-70-374m1-14 (open squares). The standard deviations for each point were less than 4%. D, SWAP-70-5 after cultivation for 7 days. E, SWAP-70-374-2 after cultivation of 5 days. F, SWAP-70-374-24 after cultivation for 7 days. Error bars indicate standard deviation.

Generally, transformed cells grow faster than the normal cells, pile up and do not exhibit contact inhibition. Therefore, whether MEFs expressing SWAP-70-374 show contact inhibition and resistance to nutrient starvation was tested. The transformants were cultured without medium change for 2 weeks. During the cultivation, parental and wild-type SWAP-70-expressing MEFs reached confluence and survived more than one week (Fig. 6A and D). In contrast, those expressing SWAP-70-374 grew faster but when nutrients were used up, they died quickly (Fig. 6B, E, F). These results suggest that MEFs expressing SWAP-70-374 are not able to stop growing by contact inhibition and lose resistance to nutrient starvation. Those expressing SWAP-70-374m1 survived under the condition of nutrient starvation (Fig. 6C). Therefore, expression of SWAP-70-374 gives some characteristics of cell transformation to MEFs.

### Sanguinarine inhibits overgrowth of SWAP-70-374-transformed cells as well as membrane ruffling induced by stimulation with EGF

**Figure 7:**
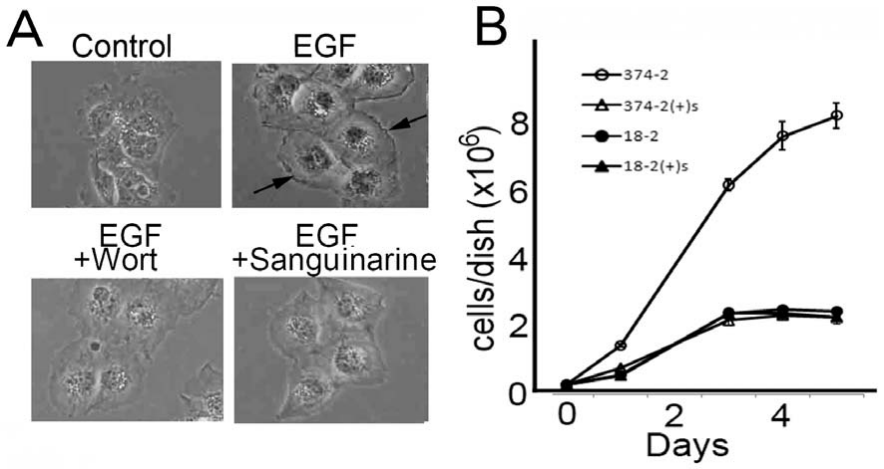
Sanguinarine inhibits membrane ruffling of Cos7 cells. A, Cos7 cells were treated with sanguinarine (2 µM) or wortmannin (100 nM) 15 min prior to EGF (100 ng/ml) stimulation for 5 min. After fixation with formaldehyde, cells were observed. The arrows indicate membrane ruffles. B, 18-2 cells or SWAP-70-374-2 cells were seeded on 6-cm dishes at a cell density of 1×10^5^ cells in the dish. Cells were grown in the presence or absence of sanguinarine (0.8 µM) with medium change on every other day. Cell numbers were monitored. Open circles: SWAP-70-374-2 cells without sanguinarine; closed circles: 18-2 cells without sanguinarine; open triangles: SWAP-70-374-2 cells with sanguinarine, closed triangles: 18-2 cells with sanguinarine. Error bars indicate standard deviation.

To understand how SWAP-70-374 transforms MEFs, drugs that inhibit overgrowth of SWAP-70-374-transformed cells under nutrient starvation conditions were screened. Among 300 drugs that include inhibitors for signaling molecules, proteins required for secretion systems, and various kinases, 5 drugs exhibited such an effect. To find which of these drugs target SWAP-70 itself or something very closely related to the SWAP-70 pathway, another assay was performed with these drugs. It has been shown that SWAP-70 is required for membrane ruffling induced by EGF, which is inhibited by wortmannin, a PtdIns-3 kinase inhibitor (Fig. 7A). The selected drugs were added to this system. Only sanguinarine inhibited membrane ruffling of Cos7 cells induced by EGF stimulation (Fig. 7A). Therefore sanguinarine inhibits two functions of SWAP-70, which is membrane ruffling and cell transformation. These results suggest that this drug targets SWAP-70 itself or another factor which is closely related to the SWAP-70 pathway. Sanguinarine greatly inhibited growth rate of SWAP-70-374-transformed cells by sanguinarine but not that of parental MEFs (Fig. 7B). These results support the idea that sanguinarine blocks the SWAP-70-related signaling pathway.

### ERK1/2 is activated in SWAP-70-374-transformed cells

**Figure 8:**
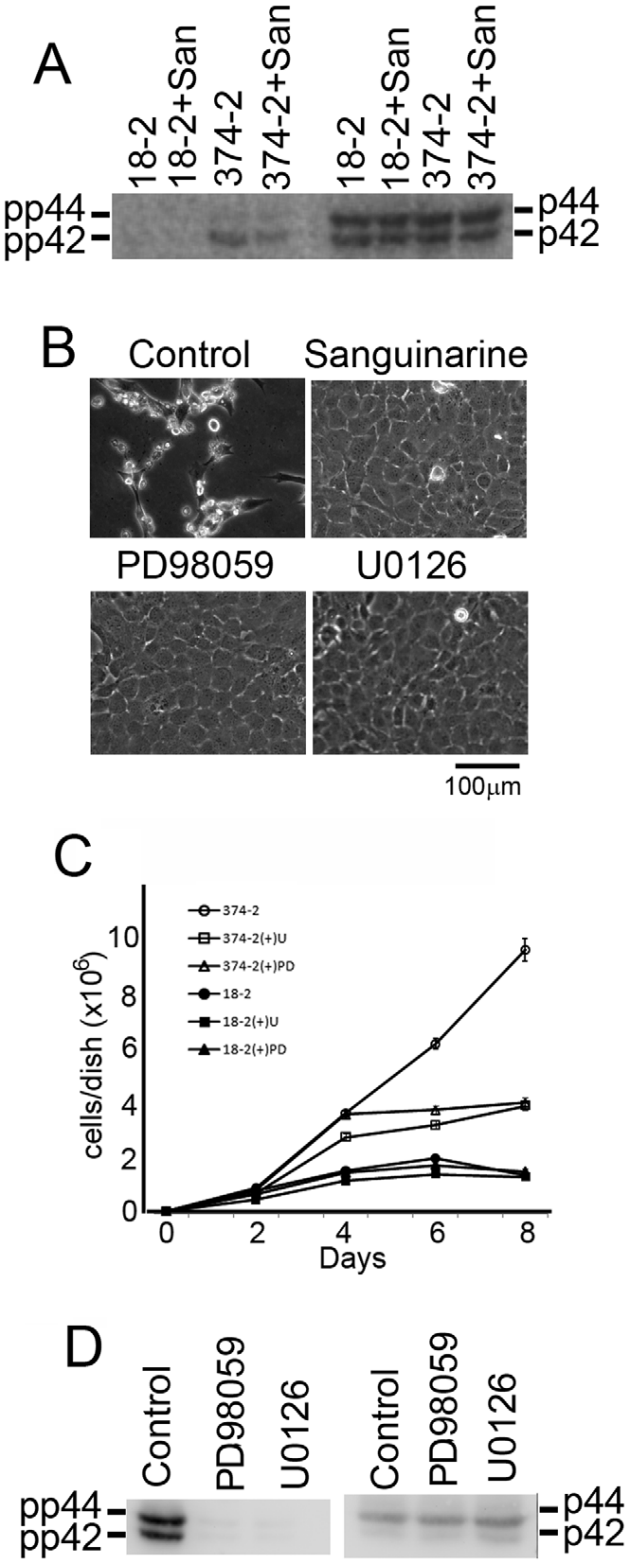
Effect of sanguinarine and MEK inhibitors on growth of SWAP-70-374-transformed cells. A, A confluent culture of 18-2 and SWAP-70-374-2 cells were replated to fresh dishes and cultured for 1day in the presence or absence of sanguinarine (0.8 µM). The cells were lysed and analyzed for phosphorylation of ERK1/2. Left half, probed with an anti-phospho-ERK1/2 antibody. Right half, probed with an anti-ERK1/2 antibody. B, SWAP-70-374-2 cells (2×10^5^ cells/6-cm dish) were cultured in the absence or presence of sanguinarine (0.8 µM), PD98059 (10 µM), or U0126 (10 µM) without medium change for 7 days. In the absence of the drugs, the cells died whereas they survived in the presence of these drugs. C, 18-2 cells or SWAP-70-374-2 cells were seeded at a cell density of 2×10^5^cells per 6-cm dish. Cells were grown in the presence or absence of PD98059 (10 µM) or U0126 (10 µM) with medium change on every other day. Open circles, SWAP-70-374-2 cells without drugs; open triangles, SWAP-70-374-2 cells with PD98059; open squares, SWAP-70-374-2 cells with U0126; closed circles, 18-2 cells without drugs; closed triangles, 18-2 cells with PD98059; closed squares, 18-2 cells with U0126. Error bars indicate standard deviation. D, Confluent cultures of SWAP-70-374-2 cells were replated to fresh dishes and cultured for 1day in the presence or absence of PD98059 (10 µM) or U0126 (10 µM). The cells were lysed and analyzed for phosphorylation of ERK1/2. Left half, probed with an anti-phospho-ERK1/2 antibody. Right half, probed with an anti-ERK1/2 antibody.

To see what signaling pathway is involved in transformation of MEFs by SWAP-70-374, activation of ERK1/2, which was often seen in transformed cells, was examined. As shown in Fig. 8A, ERK1/2 was activated in SWAP-70-374-transformed cells. In contrast, this was not observed in non-transformed cells.

Therefore, the effect of MEK inhibitors on growth of SWAP-70-374-transformed cells was examined. As shown in Fig. 8B, the MEK inhibitors, PD98059 and U0126, blocked death of SWAP-70-374 expressing MEFs after starvation with nutrients as was the case with sanguinarine (Fig. 8B). In contrast, the growth rate of these cells treated with the MEK inhibitors remained unchanged until they reached confluence (Fig. 8C). This was true with the parental cell line (Fig. 8C). However, these drugs caused contact inhibition of SWAP-70-374-transformed cells, even though saturation density was higher than that of parental cell lines (Fig. 8B and C). Figure 8D shows that the MEK pathway was indeed inhibited by these inhibitors. These results suggest that activation of the ERK1/2 pathway partially contributes to transformation of MEFs by SWAP-70-374.

## Discussion

It has been shown that SWAP-70 is closely related to malignancy of cancer. Overexpression of SWAP-70 has been often seen in malignant tumor and in various tumor cell lines [1], [2]. In this paper, we demonstrate that a mutant of SWAP-70, SWAP-70-374, can morphologically transform, enhance cell growth, and abolish the ability to stop growing by contact inhibition including resistance to nutrient starvation in MEF cells. SWAP-70-374 may be a constitutively activated mutant. SWAP-70-374 was capable of binding to F-actin *in vitro*, whereas the wild-type protein was not. Corresponding to this finding, SWAP-70-374 co-localized with F-actin at the plasma membrane constitutively, when the wild-type protein did so only when cells were stimulated with growth factors.

SWAP-70-374 is likely to take a conformation similar to the activated form of SWAP-70. The finding that SWAP-70-374 can still translocate to membrane ruffles after growth factor stimulation suggests that the conformation of SWAP-70-374 may not be abnormal (Fig. 1B). ERK1/2 was found to be activated in SWAP-70-374 expressing cells, suggesting that SWAP-70-374 can activate some signaling pathways.

The localization of SWAP-70-374 to the plasma membrane was dependent on the F-actin binding activity. The PtdIns(3,4,5)P_3_ binding activity may not be required for this translocation because an additional mutation that should abolish the PtdIns(3,4,5)P_3_ binding activity did not alter the membrane localization of the protein. It has been shown that translocation of the wild-type protein to the plasma membrane from the cytosol requires the PtdIns(3,4,5)P_3_ binding activity of the protein. Therefore, the mechanism of membrane localization of SWAP-70-374 may be different from that of the wild-type protein stimulated with growth factors. These results suggest that SWAP-70-374 forms a unique complex that produces signals for abnormal growth of the cells. Although the PtdIns(3,4,5)P_3_ binding activity was not required for membrane translocation of the protein to the plasma membrane, the PtdIns(3,4,5)P_3_ binding activity appears to be still required for transformation of MEFs. Interestingly, SWAP-70-374m1, which is incapable of binding to PtdIns(3,4,5)P_3_, inhibited growth of MEFs. Expression of this protein slows down the growth of the cells and makes them flatter than the parental cells. Saturation cell density was also lower than that of the parental cells. It is likely that SWAP-70-374m1 may be in an activated conformation that can interact with other proteins in the signaling pathway: but because of SWAP-70-374m1's inability to bind to PtdIns(3,4,5)P_3,_ the signal may be stopped at SWAP-70-374m1 level.

**Figure 9:**
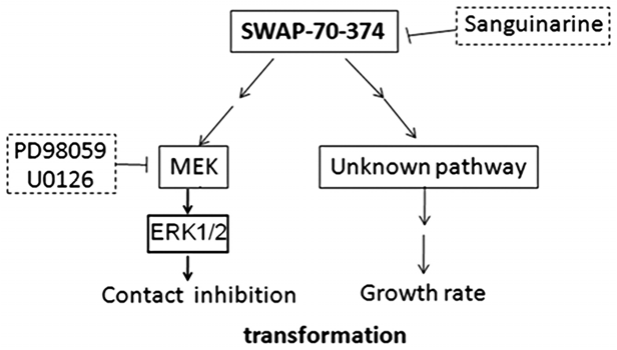
A model for signaling of SWAP-70-374-dependent transformation of MEFs. The putative signaling model of SWAP-70-374-dependent transformation is shown. So far, only the role of the ERK1/2 pathway in regulation of contact inhibition is known: there should be at least one more signaling pathway responsible for fast growth of the cells.

Sanguinarine has been shown to exhibit anti-tumor activity. It has been shown that sanguinarine induces apoptosis on various tumor cell lines [10], [11], [12], [13], [14]. It has been suggested that sanguinarine interacts with chromatin to modulate transcription [16]. In this paper, sanguinarine was found to inhibit transformation of MEFs by SWAP-70-374. Only SWAP-70-374-dependent acceleration of growth rate was inhibited by sanguinarine. Growth rate of 18-2 was not affected by sanguinarine. This can be explained as follows. SWAP-70-374 may activate not only the ERK1/2 pathway but also some other signaling pathways. One of the unknown pathways may be responsible for acceleration of the growth rate (Fig. 9). This unknown pathway may not be activated in 18-2 cells. In such a case, sanguinarine cannot inhibit growth of 18-2 cells but can inhibit that of SWAP-70-374-transformed cells (Fig. 7B). Unlike sanguinarine, MEK inhibitors did not slow down the growth of SWAP-70-374-transformed cells (Fig. 8C). However, MEK inhibitors caused restoration of contact inhibition of these cells (Fig. 8C). These results suggest that the ERK1/2 pathway regulates only contact inhibition of these cells and that there may be at least one other pathway that stimulates cell growth (Fig. 9). Characteristics of MEFs may differ from lines to lines. Whether the regulation of contact inhibition by the ERK1/2 pathway is common to all the MEF lines should be tested in the future.

Sanguinarine also inhibited membrane ruffling of Cos7 cells after growth factor treatment, which is another function of SWAP-70. Therefore, it is possible that this drug may inhibit SWAP-70-mediated signaling pathways. In addition to sanguinarine's role in the nucleus, our new finding may supply information important for understanding of the function of sanguinarine. It should be noted that the concentration of sanguinarine required for inhibiting SWAP-70-374-induced transformation was much lower than that required for inhibition of chromatin related reactions.

Because PtdIns(3,4,5)P_3_ binding activity of the SWAP-70 mutant is required for transformation of MEFs, it is likely that the PtdIns 3-kinase pathway is also involved in the transformation of MEFs by the mutant SWAP-70. However, which signaling pathways other than the ERK1/2 pathway are required for cell transformation are not known. Further examination may be required to understand the whole picture of signaling pathways activated by the SWAP-70-374-F-actin complex.
